# Experience-Driven Differences in Childhood Cortisol Predict Affect-Relevant Brain Function and Coping in Adolescent Monozygotic Twins

**DOI:** 10.1038/srep37081

**Published:** 2016-11-22

**Authors:** Cory A. Burghy, Michelle E. Fox, M. Daniela Cornejo, Diane E. Stodola, Sasha L. Sommerfeldt, Cecilia A. Westbrook, Carol Van Hulle, Nicole L. Schmidt, H. Hill Goldsmith, Richard J. Davidson, Rasmus M. Birn

**Affiliations:** 1 Center for Healthy Minds at the Waisman Center, University of Wisconsin-Madison, 1500 Highland Avenue, Madison, WI, 53705, USA; 2 Waisman Laboratory for Brain Imaging and Behavior, University of Wisconsin-Madison, 1500 Highland Avenue, Madison, WI 53705, USA; 3 Department of Medical Physics, University of Wisconsin-Madison, 1111 Highland Avenue, Madison, WI, 53705, USA; 4 Waisman Center, University of Wisconsin-Madison, 1500 Highland Avenue, Madison, WI 53705, USA; 5 Department of Psychology, University of Wisconsin-Madison, 1202 West Johnson Street, Madison, WI 53706-1969, USA; 6 Department of Psychiatry, University of Wisconsin-Madison, 6001 Research Park Blvd., Madison, WI 53719, USA

## Abstract

Stress and emotion involve diverse developmental and individual differences. Partially attributed to the development of the prefrontal cortex (PFC), the amygdala, and hypothalamic-pituitary-adrenal axis, the precise genetic and experiential contributions remain unknown. In previous work, childhood basal cortisol function predicted adolescent resting-state functional connectivity (rs-FC) and psychopathology. To parse experience-driven (non-genetic) contributions, we investigated these relations with a monozygotic (MZ) twin design. Specifically, we examined whether intrapair differences in childhood afternoon cortisol levels predicted cotwin differences in adolescent brain function and coping. As expected, intrapair differences in childhood cortisol forecast amygdala-perigenual PFC rs-FC (*R*^2^ = 0.84, FWE-corrected *p* = 0.01), and amygdala recovery following unpleasant images (*R*^2^ = 0.40, FWE-corrected *p* < 0.05), such that the cotwin with higher childhood cortisol evinced relatively lower rs-FC and poorer amygdala recovery in adolescence. Cotwin differences in amygdala recovery also predicted coping styles. These data highlight experience-dependent change in childhood and adolescence.

Stress sensitization involves a constellation of genetic and experience-driven processes. Considerable study has focused on how and when the function of the hypothalamic-pituitary-adrenal (HPA-) axis relates to neural behavior and psychopathology^1^, specifically, how basal cortisol levels predict limbic and prefrontal cortex structure and function^2^, and whether that variance predicts individual differences in how people approach, cope with, and recover from unpleasant/stressful experiences^3^,^4^,^5^. However, separating heritable, genetic influences from environmental, experience-driven contributions, to explore aspects of plasticity or loci for intervention, is a daunting task. To investigate these experiential differences, we use a longitudinal monozygotic (MZ) twin design. Studying differences between MZ cotwins offers key advantages for detecting environmental effects on affect-related phenotypes, given that MZ twins share ≫99% of their genomic DNA base sequences.

## Stress and Cortisol

Studies of both individuals and twins have focused on genetic and environmental contributions to changes in basal cortisol levels measured across the day. Morning and waking cortisol levels have been closely tied to heritable genetic factors, explaining roughly 60–80% of the variance in basal levels, whereas later afternoon/evening cortisol levels appear to be more sensitive to experience^6^. Furthermore, higher levels of basal cortisol late in the day have been reliably related to later internalizing symptoms in childhood^3^,^7^ and adolescence^2^, and to atypical patterns of neural activation during emotion regulation in adults^8^. Thus, afternoon and early evening basal cortisol levels may be most relevant to stress sensitization in childhood, such that those children evincing relatively high basal cortisol levels cope less well with stress. Poor emotional recovery may be reflected in neurobiological developmental differences in the brain as well^9^,^10^.

Glucocorticoid receptor density in the ventromedial prefrontal cortex (vmPFC) and limbic system, specifically the amygdala and hippocampus, has been proposed as a possible mechanism for environmental stress-cortisol-brain interactions relevant to psychopathology, as evinced in rodent research^11^,^12^. Human work has further highlighted potential sensitivity in the amygdala-vmPFC pathway for emotional reactivity and recovery^13^,^14^,^15^. Recent research detected a possible developmental stress cascade beginning with early life stress exposure in infancy, leading to symptoms of anxiety and depression in adolescence via childhood evening basal cortisol and adolescent brain function^3^. Higher cortisol and reduced amygdala-vmPFC rs-FC were both suggestive of poor recovery and mediated robust relations between higher childhood basal afternoon cortisol and adolescent anxiety in girls. Such gender differences in how cortisol function and psychopathology relate have been detected previously in childhood, and these differences may reflect varying developmental pathways in boys versus girls. That is, the HPA-axis in girls might be more sensitive to fluctuations in stress during development. This greater sensitivity might then be reflected in more anxious symptoms in girls^7^.

Despite progress in understanding how the HPA-axis is coupled with neural correlates of emotional behavior, we do not understand which features of neural activity are modulated by experience and/or genetics, and how or when this modulation occurs^1^,^3^,^4^. If these relations are indeed sensitive to experience, they may be associated with coping styles. Coping styles influence the interpretation of stressors and how stress is experienced and resolved, and these differences may be partially-reflected neurally in the ventral PFC^16^. For example, denial coping may be adaptive in the short term, but it predicts harmful longer-term consequences on well-being. In contrast, acceptance coping is linked to lower internalizing symptoms^17^, and it mediates affect dysregulation in the children of mothers who favor this type of coping^18^.

## The Monozygotic Cotwin Difference Approach

The key biological fact is that MZ cotwins are identical for DNA sequence variants with the exception of rare somatic mutations. MZ cotwins can differ in epigenetic modifications, and these differences tend to increase over the lifespan^19^. Thus, by studying the relative difference between cotwins, we can target experiential differences while holding many non-genetic factors (e.g., age, parenting, socioeconomic effects) quasi-constant. This design can reveal whether a measured experience or exposure is truly environmental/experience-driven (i.e., unconfounded with genotype) whereas other designs are unable to exclude the possibility that measured experiences and exposures are veiled indicators of genetic differences or susceptibilities.

The recognition of this set of considerations has led to an upsurge of MRI research using variants of the MZ difference design^20^. Earlier studies that examined neural differences in MZ twin pairs selected as discordant for psychopathology (attention deficit hyperactivity disorder^21^,^22^; anxiety and depression^23^) sought neural differences of environmental origin, generally without measuring an earlier putative source or marker of these environmental differences. In this study, we ask whether late day cortisol levels during childhood affect adolescent neural function and coping style in a manner that is environmentally mediated. Targeting functional rather than structural brain differences may prove powerful given that functional neural measures may be more sensitive to environmental effects.

The brains of MZ cotwins show gross morphological similarity^24^. In cross-sectional neuroimaging studies involving non-twins, morphological differences between participants complicate the interpretation of functional findings, as underlying structure varies widely. By examining brain function and behavior in MZ cotwins who vary in concordance on measures of basal afternoon cortisol function in childhood, we can determine which brain circuits are associated with variations in cortisol function and, by proxy, stress sensitization/regulation. Although genetic influences on stress and affect regulation are substantial^25^,^26^, the MZ twin difference design affords a unique opportunity to identify patterns of brain function that exclusively reflect experience-dependent influences. The circuits identified in this way may be the most relevant to understanding how psychosocial factors get “under the skin” and impact the brain in ways that influence the development of psychopathology.

## Study Design and Hypotheses

We used the MZ cotwin difference design to interrogate influences on the development of individual differences in afternoon basal cortisol levels in childhood and how they predict brain function at rest and in response to unpleasant visual stimuli during adolescence. We also examined how these changes may be reflected in adolescent cognitive coping styles. Drawing from a larger birth record-based twin sample followed since early childhood, we asked how MZ intrapair differences in afternoon basal cortisol at age 7.5 years predicted intrapair variations in adolescent neural function and behavior almost a decade later in a selected subsample of 13 twin pairs (at mean age 15.83 years, *SD* = 1.67 years). Cortisol was collected over three days between 4 pm and 6 pm to maximize basal cortisol function sensitive to environmental input^6^. We examined neural function in two ways, using voxel-wise linear regressions of both amygdala-seeded rs-FC and neural recovery to unpleasant images^27^,^28^. Acceptance and denial coping styles were indexed with the COPE Inventory^29^ at the time of the imaging session. Finally, cotwin difference scores for each measure were calculated by subtracting the twin with lower childhood afternoon basal cortisol levels from the cotwin with higher cortisol levels. This method allows us to infer directionality in detected associations between childhood cortisol and later adolescent measures of brain function and behavior.

Our aims were threefold: to examine the predictive power of MZ intrapair differences in early neuroendocrine function on, (1) adolescent resting-state functional connectivity, (2) neural recovery during an affective task, and (3) to assess the associations among early cortisol, rs-FC, task estimates of neural recovery and concurrent coping styles. Our central prediction was that the cotwin with the relatively higher afternoon childhood basal cortisol level would have lower adolescent rs-FC between amygdala and PFC, particularly in the vmPFC region^2^. Following from this, we also expected that the cotwin with higher childhood afternoon basal cortisol level would also show a prolonged amygdala response to unpleasant (compared to neutral) stimuli in a task where participants were asked to passively view unpleasant and neutral images paired with neutral faces. Recovery following a negative emotional image should be associated with diminished limbic activation, particularly in the amygdala’s response to a neutral face following the picture. Thus, we hypothesized that cotwin differences in childhood cortisol would lawfully relate to, and be most pronounced during, recovery in negatively valenced trials paired with neutral faces. Poorer recovery from the unpleasant image in these trials should lead to subsequent affective coloring of the neutral face stimuli^28^.

Moreover, we predicted that intrapair differences in amygdala recovery would be associated with concurrent coping strategies. Specifically, we expected cotwins with relatively longer amygdala recovery would engage in less efficient coping strategies (e.g., denial), given their lack of success in automatically regulating negative affect. Thus, this amygdalar persistence would reflect a broader disruption in both cognitive and behavioral skill in regulation, and would be inversely correlated with more adaptive regulatory strategies (e.g., acceptance). Finally, to explore whether these relations were specific to intrapair differences, we constructed and ran a series of 10,000 two-tailed linear regressions with randomly assigned cotwin pairs (i.e., pseudo-twins from the current sample; same-sex pair restricted). In these analyses, the *r*^2^ value of the actual twin pair based regression model was compared against the *r*^2^s of the pseudo-twin pair regressions, and the resultant *percentages* presented below reflect the probability of detecting the highlighted differences by chance (i.e., if the actual pair’s score falls under 1%, this reflects a likelihood of getting such an association by chance as less than 1%).

## Results

### Childhood Afternoon Basal Cortisol

Prior to further analyses, a paired-samples t-test was performed to examine intrapair differences in childhood cortisol function, to determine whether cotwins differed significantly, which is important for interpreting subsequent analyses. As expected, the higher versus lower cotwins significantly differed in cortisol function, *t*(32) = 4.42, *p* = 0.001.

### Resting-State Functional Connectivity and Cortisol

**Figure 1:**
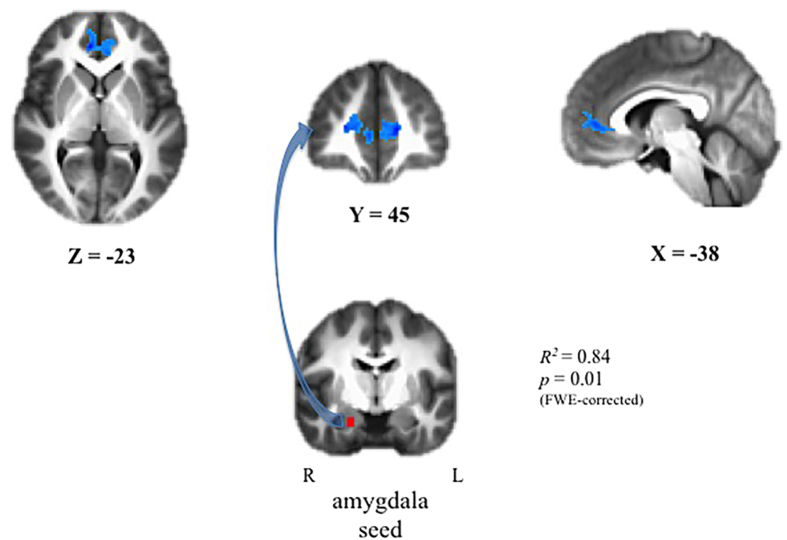
MZ cotwin differences in late afternoon cortisol at child age 7.5 yrs and resting-state functional connectivity between the right amygdala and pgPFC at 15.5 yrs. Intrapair differences in rs-FC between the right amygdala and pgPFC are significantly negatively associated with childhood cortisol (*t*(12) = 7.94, *R*^2^ = 0.84, FWE-corrected *p* = 0.01). Blue hues reflect a heat map of voxelwise connectivity within the surviving cluster, where darker hues of blue reflect reduced connectivity.

Significant associations between childhood afternoon basal cortisol and adolescent rs-FC were detected using both amygdala seeds, and no statistically significant laterality effects were observed, Fisher’s *z* = −0.47, NS. The findings using the right amygdala seed are presented here. As predicted, intrapair differences in childhood afternoon cortisol predicted intrapair differences in adolescent amygdala-PFC rs-FC. The connectivity patterns were present in the amygdala-pgPFC pathway (*t*(12) = 7.94, *R*^2^ = 0.84, FWE-corrected *p* = 0.01), such that the cotwin with relatively higher cortisol in childhood evinced lower connectivity than their cotwin (Fig. 1). Pseudopairing analyses bolstered these results (the model using the actual pairs fell below the 1^st^ percentile of the potential pseudopaired *r*^2^s).

### Behavioral Effects of Preceding Picture on Face Likability

To test for emotional perseveration following image-offset of negative and neutral images, we performed paired t-tests to examine differences (ordered by early cortisol levels as above) in the likability of faces following negative versus neutral images in both the 1 s and 3 s delay conditions. No significant differences were observed in the 1 s delay trials; thus, those trials were not included in further analyses. In the 3 s delay trials, between-subjects analyses revealed that faces following negative images (M = 39.69, SD = 14.68) were rated as less likable than faces following neutral images (M = 43.39, SD = 13.21; *t*(32) = −3.72, *p* = 0.04). Faces following negative images were less likable than the set of novel faces (M = 42.24, SD = 13.78; *t*(32) = −3.43, *p* = 0.04).

Turning to the examination of cotwin differences in likability, cotwin differences in cortisol predicted how adolescents rated faces. The cotwin with relatively higher childhood afternoon basal cortisol rated faces paired with negative pictures (M = 35.56, SD = 15.45) as less likable than their cotwin (M = 44.26, SD = 15.76; *t*(32) = −2.22, *p* = 0.05). These data suggest that the processing and encoding of these faces was affectively colored by the preceding image and varied based on the cortisol status of each cotwin.

### Task-Evoked Neural Recovery and Cortisol

**Figure 2:**
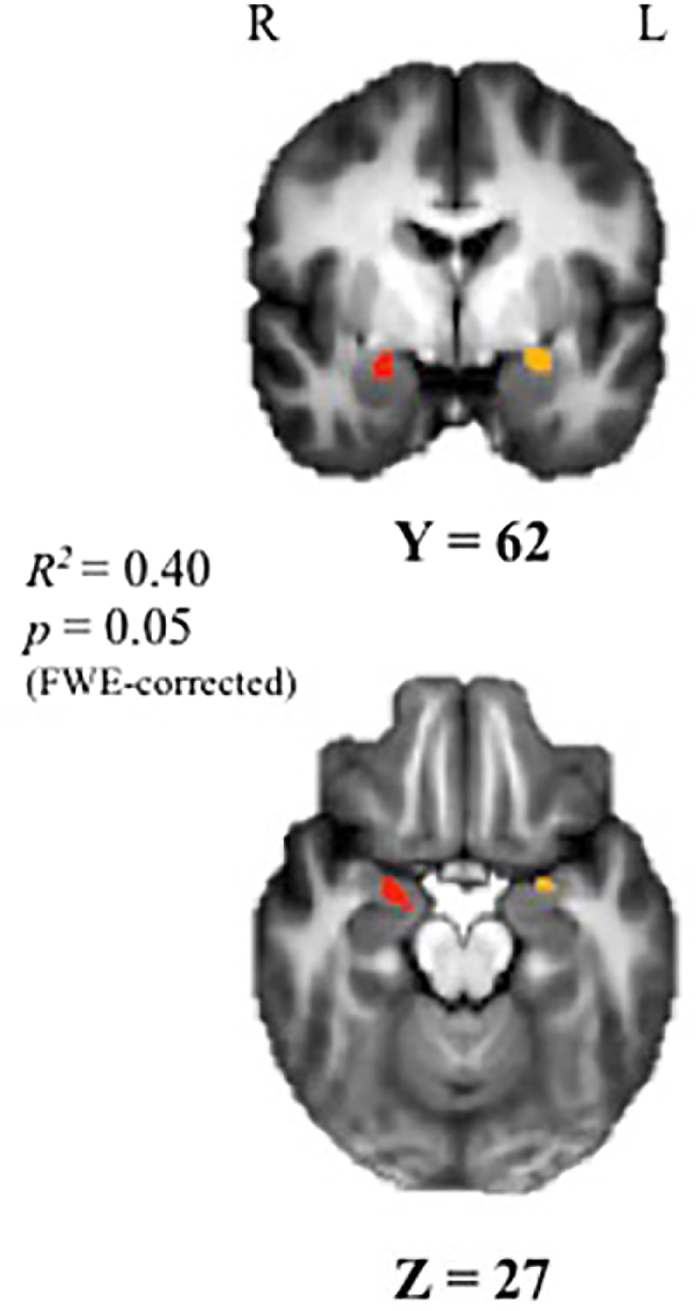
Correlation between MZ cotwin differences in late afternoon cortisol at child age 7.5 yrs and task-evoked right amygdala activity during modulated recovery from unpleasant vs neutral images paired with neutral male faces indexed at 15.5 yrs. Voxelwise analyses revealed that intrapair differences in childhood afternoon basal cortisol significantly predicted bilateral amygdala during the recovery period (*t*(12) = 4.34, *R*^2^ = 0.40, FWE-corrected *p* = 0.05). The right amygdala cluster is depicted in red, and the left in yellow.

Intrapair differences in childhood cortisol predicted bilateral cotwin disparities in amygdala-modulated recovery voxelwise (*t*(12) = 4.34, *r*^2^ = 0.4, FWE-corrected *p* = 0.05; Fig. 2). Cotwins with relatively higher afternoon cortisol evinced prolonged amygdala activation, suggesting poorer recovery from the unpleasant image. Using a Pearson bivariate correlation (*r*), we examined cotwin differences between the extracted amygdala-pgPFC rs-FC estimates and right amygdala recovery seed. Here, poorer amygdala modulated recovery (i.e., higher PSC) was significantly associated with reduced amygdala-pgPFC rs-FC (*r* = −0.66, *p* = 0.014). Pseudopair analyses supported these detected associations (2^nd^ percentile).

### Affect-Relevant Brain Activity and Coping Style

**Figure 3:**
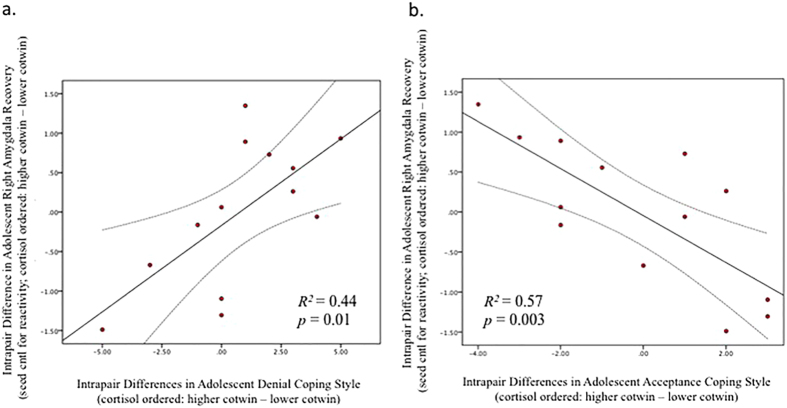
Bivariate correlations between MZ cotwin differences in (**a**) denial and (**b**) acceptance coping behaviors with task-evoked right amygdala seed activity during recovery (while controlling for initial reactivity) from unpleasant vs neutral images paired with neutral male faces indexed at 15.5 yrs. Intrapair differences in denial coping behaviors were significantly *positively* correlated with right amygdala activity during the recovery period (*R*^2^ = 0.44, *p* = 0.01), while cotwin differences in acceptance coping behaviors were *inversely* correlated with differences in right amygdala activity during recovery (*R*^2^ = 0.57, *p* = 0.003). Error lines represent confidence intervals at 95%.

Cotwin differences in amygdala recovery were significantly associated with both self-reported styles of coping. Specifically, cotwins with poorer modulated recovery to unpleasant images reported relatively higher denial-type coping behaviors (*r* = 0.67, *p* = 0.013; Fig. 3a), and were also less likely to report acceptance coping strategies (*r* = −0.75, *p* = 0.003; Fig. 3b). This combination of findings supports the hypothesis that, within twin pairs, cotwins with less efficient neural recovery engage in less efficacious coping strategies. The detected MZ intrapair relations between coping styles and amygdala recovery model fell in the 9^th^ and 1^st^ percentiles, respectively, of potential pseudopaired *r*^2^s, consistent with such variation originating from experience-dependent causes.

## Discussion

Using functional brain imaging with MZ twins, we show that intrapair differences in childhood cortisol predict neural rs-FC between the amygdala and pgPFC as well as amygdala recovery during an implicit emotion regulation task in adolescence. Intrapair differences in childhood cortisol also predicted behavioral measures of affective preservation, indexed by the likability of neutral faces presented following unpleasant images. Finally, cotwin differences in amygdala recovery significantly predicted the types of coping strategies individuals endorsed. Our results underscore the power of the MZ twin difference design to uncover robust longitudinal associations between early hormonal variation and both adolescent brain function and coping strategies over a near-decade span. Furthermore, these detected relations can be causally attributed to environmental influences that are not shared by MZ cotwins growing up in the same family.

Intrapair differences in afternoon cortisol levels in childhood, measured using salivary cortisol over a three-day period, significantly predicted experience-driven differences in amygdala-pgPFC rs-FC, a crucial pathway for affective processes and stress regulation in adolescence^14^. These findings extend our prior work and provide initial data to answer one of our lingering questions regarding the nature of associations between cortisol and rs-FC in development. Although this cluster is slightly superior to the vmPFC cluster detected in previous work^2^, both the pgPFC and the vmPFC are implicated in the regulation and the experience of negative emotion, and both possess direct, bidirectional connections with the amygdala^15^. Thus, we believe that the detected pathway is grounded in relevant anatomical loci in both cases. The plastic nature of these sectors of the PFC in development may partially explain the variance in loci; however, future work should be dedicated to the delineation of these associations.

Intrapair differences in childhood cortisol also predicted more amygdala activity during a post negative picture modulated recovery epoch. Basal afternoon/evening cortisol levels are more environmentally-mediated than other metrics of cortisol function^3^,^6^ and are postulated to reflect poorer recovery to the stresses and challenges of the day. This lack of recovery appears to echo in the adolescent’s amygdala function nearly a decade later. The amygdala plays a central role in the instantiation of negative affect, and its activity diminishes as a function of effective regulation of negative emotion^8^. These conclusions are bolstered by behavioral face likability data, which demonstrate that individuals rated faces paired with unpleasant images more negatively than those paired with neutral images or novel faces. Furthermore, the extent to which individuals rated faces as less likable when paired with unpleasant images versus novel faces, was also predicted by higher childhood cortisol levels. Taken together, these findings suggest that these adolescents perseverated in their affective responses to unpleasant images in a manner somewhat analogous to the way that their basal cortisol levels in childhood remained elevated late into the day.

In fact, correlations between intrapair differences in amygdala recovery and coping styles supported this theme that cotwins who were relatively sensitized to stress (e.g., had poorer amygdala recovery) were also likely to engage in less effective coping strategies such as denial coping in adolescence. Specifically, twins with greater MR signal in the amygdala during the recovery period were more likely to report engaging in *denial* coping, and less likely to engage in more adaptive *acceptance* coping behaviors than their more resilient (lower cortisol, faster recovery) cotwins. These data are consistent with previous work in pain processing and regulation, where acceptance and denial coping styles predicted opposing engagement of the ventrolateral PFC (vlPFC) during uncontrollable vs. controllable pain stimuli, suggesting differential engagement of affect-relevant circuitry^16^. Not only do the current data suggest that neural indices of emotional recovery map well onto individual differences in coping, they also provide potential loci for cognitive behavioral intervention targeting how and when individuals appraise stressors, whether they are controllable or not, and possible actions they may take to cope with them.

The use of the intrapair MZ cotwin difference design uncovers powerful relations between individual differences in phenotypic features of stress response and neural behavior in both resting and task-related brain function. The magnitude of associations between these variables is considerably greater than has been typically reported in genetically unrelated singletons^30^. Removing extraneous variance associated with background genetics, as well as a host of other factors such as socioeconomic variation, apparently reveals stronger associations between brain function and biobehavioral phenotypes. Moreover, this design permits us to unambiguously conclude that the variation in brain function and behavior is experience-dependent. These findings are broadly consistent with other human studies using MZ twins^19^ and rodent studies using genetically identical clones^31^ that indicate that as genetically identical individuals begin to explore their environments and undergo developmental change, robust experience-dependent epigenetic variation is induced.

Limitations of this work include our small sample size, which precludes exploring gender differences. A larger sample of MZ cotwins within a more restricted age range would also be valuable, as would longitudinal imaging data on the same set of MZ cotwins. In addition, we cannot speak to levels of psychopathology, but rather focus on task-derived behavior and coping styles as possible mechanisms by which vulnerability to psychopathology might be generated. Future work in this area would benefit from a more exhaustive survey of symptom data from MZ cotwins, both in terms of breadth and age range. For example, although we can now conclude that the detected differences are experience driven, more basal cortisol and imaging data taken at several points during development may uncover the rate at which these changes occur and how they more precisely relate in development.

Finally, these findings underscore the importance of experience, particularly early experience, in the development of prefrontal-amygdala circuitry by illuminating where experience-dependent variation in this circuitry is related to affective recovery processes and provide support for the examination of these processes in childhood as possible loci for intervention or training in coping and emotion regulation skills that would afford children more adaptive patterns of stress responding.

## Methods

### Participants

Participants were a subsample of 35 MZ twin pairs from the Wisconsin Twin Project^32^, a large longitudinal study of twins followed since childhood. Assessments of this sample began at approximately age 7 years, and imaging data were collected between the ages of 13–19 years (*M*_*age*_ = 15.83 years, *SD* = 1.67 years). Monozygosity was confirmed with genetic testing. Twelve twin pairs were excluded from analyses due to MRI incompatibility and/or history of neurological disorder. One additional pair was excluded from task analyses due to motion, while an additional 4 pairs did not complete resting-state scans. Of the remaining twin pairs, 13 (6 female pairs) had cortisol data. The final sample was 75% non-Hispanic White, and 67.6% right-hand dominant. Informed consent (and parental permission in childhood) was obtained for all assessments, and participants received monetary compensation. University of Wisconsin–Madison Institutional Review Boards approved all procedures. All methods were carried out in accordance with the approved guidelines.

### Childhood Afternoon Cortisol

Childhood basal cortisol (*M*_*age*_ = 7.5 years) was collected over three days. Parents were instructed to collect salivary samples at a target time between 4:00 pm and 6:00 pm. In the current subsample, 95% complied with the prescribed time frame and 19% of children were taking over-the-counter (OTC) medications (e.g., ibuprofen, cold medicine). Samples were assayed for cortisol in duplicate using a radioimmunoassay modified for saliva (Pantex, Santa Monica, CA). Two non-blind internal controls were included in each assay. For the low control, the average value was 0.082 μg/dL with inter- and intra-assay Coefficient of Variations (CVs) of 7.2% and 6.1%, respectively. For the high control, the average value was 0.84 μg/dL with inter- and intra-assay CVs of 8.1% and 5.3% respectively. Results were considered acceptable if the CV of the duplicate samples was <20%. Participants were included if they provided sufficient, uncontaminated samples on at least 2 of 3 days. Mean levels were normalized via log transformation and residualized for time of collection and OTC medication usage.

### Imaging Data Acquisition and Processing

Structural and functional images were collected on a 3 T MRI scanner (Discovery MR750, General Electric Medical Systems, Milwaukee, WI, USA) with an 8-channel RF head coil array. T1-weighted structural images (1 mm^3^ voxels) were acquired axially with an isotropic 3D Bravo sequence (TE = 3.18 ms, TR = 8.13 ms, TI = 450 ms, flip angle = 12°). T2*-weighted gradient-echo echo-planar pulse sequence images were collected during resting state and the task with TE = 25 ms, TR = 2000 ms, and flip angle = 60°. The resting-state scan was 420 s (210 volumes), and each of the five task scans was 530 s (265 volumes). Functional volumes had a resolution of 3.5 × 3.5 × 5 mm^3^ (matrix size = 64 × 64; 30 sagittal slices). Most data reduction steps were performed using AFNI^33^. Corrections made on the functional data included slice time shift correction, rigid body volume registration, and 12-parameter affine alignment to the T1-weighted anatomical images using a Local Pearson correlation cost function^34^. Anatomical images were segmented with SPM8 (Wellcome Department of Cognitive Neurology, UCL, UK) and warped to a common group-space using DARTEL^35^. The anatomical images for each participant were averaged together to make a group average template, which was subsequently normalized to MNI space to create a transformation matrix for use when warping functional data to MNI space. The first four volumes of each functional scan time-series were removed due to T1-equillibrium effects and images were transformed to MNI space and resampled to 2 mm cubic voxels.

### Resting-State Data

#### Additional image processing

FSL’s FAST automatic segmentation generated white matter (WM) and cerebrospinal fluid (CSF) masks from the anatomical images^36^,^37^. The average signal intensity time-course of the functional data within the eroded WM and CSF masks, their first derivatives, and the six motion registration parameters were taken as signal of no interest and regressed from data^38^. Time points where the summed squared difference (SSD) from successive time points of the estimated motion parameters exceeded 1 mm were censored in this nuisance regression. The functional image time series were temporally band pass filtered (0.01 Hz < f < 0.1 Hz^38^) and spatially smoothed with a 3D Gaussian kernel (FWHM = 6 mm).

#### Functional connectivity

RS-FC estimates were computed using a seed-region-based approach^39^,^40^. Binary masks of the left and right amygdala were created using 4 mm spheres generated around the central voxel of each Talairach Daemon ROI^41^. The average pre-processed fMRI signal intensity time-course over each amygdala was then regressed against the signal intensity time-courses voxelwise using ordinary least squares regression. Between-twin differences in the rs-FC were computed by subtracting the Fisher z-transformed FC map of the twin with the lower level of cortisol from the twin with the higher level of cortisol, as described above. Intrapair difference rs-FC maps were then entered into two-tailed regressions while co-varying behavioral variables of interest. Multiple comparison correction was performed by setting a minimum cluster size based on α significance values less than or equal to 0.05, which incorporates the estimated spatial smoothness.

### fMRI Task

#### Task design

**Figure 4:**
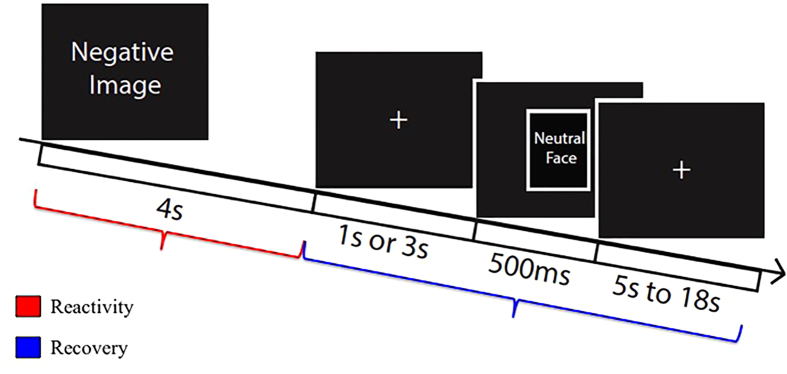
Trial schematic for the uninstructed emotion regulation task. Unpleasant, neutral, or pleasant pictures were presented for 4 s. In two-thirds of the trials, picture offset is followed by a variable inter-stimulus interval of 1 or 3 s prior to a 500 ms neutral male face presentation. Inter-trial intervals varied from 5 s to 18 s.

Participants completed a passive picture-viewing task as part of the MRI session used in previous work to index emotional reactivity and recovery^28^. The task consisted of 180 trials (60 positive, negative, and neutral images) and was divided over 5 blocks. All images were drawn from the International Affective Picture Set (IAPS^27^) and selection criteria are as follows: negative images had valence ratings of less than 4 (range = 1.38–3.72) and arousal ratings greater than 3.5 (range = 3.67–6.62). Neutral images had valence ratings in the range of 4.38–5.53, and arousal ratings of less than 4 (range = 2.77–3.76)(see Table 1 for a full list of images, means, and standard deviations). These criteria for neutral images were identified in order to select images whose valence and arousal range overlapped as little as possible with negative images and were neither too unpleasant or pleasant, and non-evocative (low arousal). In each trial, a white fixation cross was displayed in the center of a black screen (1 s), followed by a picture (4 s). Valence and image order were randomized within the task. Participants were instructed to indicate the valence of each image as negative, neutral, or positive via button press as images appeared. Following picture offset, a second fixation screen was presented, and participants were told to maintain their gaze on the fixation cross for the entire duration of the trial. In two-thirds of the trials, images were followed by a neutral male face (500 ms) either at 1 or 3 s post-picture offset (see Fig. 4). The remaining trials had no face presentation. After the offset of face images, the fixation cross was re-presented with an inter-trial interval (derived from an exponential distribution) that varied from 5.5 to 17.6 s (*M* = 8.89 s), providing sufficient variation to estimate the evoked BOLD response function. Each face was presented twice and consistently paired with a valence and delay (1 or 3 s) within participants (randomized between subjects).

The faces served as a behavioral measure of post-image recovery. Adult male faces were chosen from several validated face sets^42^,^43^,^44^. In order to look at effects of the preceding image on likability of the neutral faces, participants were asked to complete likability ratings of the 60 male faces they had seen during the task and a set of 60 age-matched unfamiliar faces 1 hour post-scan (randomly ordered). Participants were not verbally reminded which faces they had previously seen or with which image they were paired. Participants rated likability of each face on a continuous scale from 0 (Really Dislike) to 100 (Really Like), where a 50 rating reflects that the participant neither likes nor dislikes the face. The ratings were completed in the laboratory using E-Prime 2.0 software (Psychology Software Tools, Pittsburg, PA).

#### Image processing and analysis

The functional task data from individual subjects were modeled using 5 sine functions to estimate the hemodynamic response. To examine reactivity and recovery effects, each trial was separated into two six-second epochs beginning at IAPS image onset. These epochs were chosen to divide the average trial into roughly two equal blocks, with the first epoch representing the initial response to the image (reactivity), and the second to represent both the neural recovery to the image and response to the face presentation where applicable. Thus, the recovery epoch in image + face trials are considered recovery *as modified by a neutral stimulus presentation*, or modulated recovery. Neural activity was quantified as the percent signal change (PSC) from baseline (all non-modeled data) in each epoch. To distinguish recovery from reactivity, initial PSC in the reactivity epoch was regressed onto PSC estimates of modulated recovery voxelwise prior to any valence comparisons. Picture-only recovery was calculated by subtracting recovery PSC in neutral trials from negative trials. Face trials were calculated with a double-subtraction of face and no face trials: (Negative 3 s Face–Neutral 3 s Face)–(Negative No Face–Neutral No Face, see Table 1). These contrasts were warped to MNI space, smoothed (FWHM = 6 mm), and intrapair contrasts were calculated. Both voxelwise and amygdala seed data were examined (seeds were 4 mm spheres described above in rs-FC for both right and left amygdala).

### Coping Style

Adolescent coping styles were assessed via the COPE^29^, a well-validated 60-item self-report measure of coping responses to life events and/or stressors. Based on previous research^16^, we examined Acceptance (e.g., *I learn to live with it;* Crohnbach’s *α* = 0.84) and Denial coping strategies (e.g., *I say to myself ‘this isn’t real’;* Crohnbach’s *α* = 0.83). Each item was coded on a 4-point Likert scale (1 = *I usually don’t do this at all*, and 4 = *I usually do this a lot*) and items were summed to create average scores for Acceptance (*M* = 11.39, *SD* = 2.33) and Denial (*M* = 6.21, *SD* = 2.33) coping strategies.

## Additional Information

**How to cite this article**: Burghy, C. A. *et al*. Experience-Driven Differences in Childhood Cortisol Predict Affect-Relevant Brain Function and Coping in Adolescent Monozygotic Twins. *Sci. Rep.* **6**, 37081; doi: 10.1038/srep37081 (2016).

**Publisher’s note:** Springer Nature remains neutral with regard to jurisdictional claims in published maps and institutional affiliations.

## Figures and Tables

**Table 1:** Means and standard deviations of cortisol, brain activity, coping skills, and task stimuli.

	*n*	Descriptive Statistics	
		*M*	*SD*
All Twins			
Negative Images: Valence^IAPS^	26	2.81	0.50
Negative Images: Arousal^IAPS^		5.24	0.76
Likability of faces following negative image		39.69	14.68
Neutral Images: Valence^IAPS^	26	5.02	0.76
Neutral Images: Arousal^IAPS^		3.35	0.44
Likability of faces following neutral image		43.39	13.21
Likability of novel faces		42.24	13.78
High Cortisol Cotwin	13		
Afternoon Cortisol		−0.001	0.05
Amygdala-pgPFC rs-FC		−0.09	0.12
Amygdala Recovery		.03	0.17
Amygdala Reactivity		.08	0.10
Likability of faces following negative image		35.56	15.45
Likability of faces following neutral image		41.16	13.45
Likability of novel faces		37.37	12.61
COPE: Acceptance		11.38	1.98
COPE: Denial		6.77	1.69
Lower Cortisol Cotwin	13		
Afternoon Cortisol		−0.04	0.04
Amygdala-pgPFC rs-FC		.03	0.21
Amygdala Recovery		−0.03	0.15
Amygdala Reactivity		0.01	0.11
Likability of faces following negative image		44.26	15.76
Likability of faces following neutral image		46.96	12.97
Likability of novel faces		38.24	13.67
COPE: Acceptance		11.53	1.85
COPE: Denial		6.00	2.20

Note: Cortisol is expressed in normalized units (log transformed and residualized mean μg/dL). Rs-FC estimates are correlation coefficients between the amygdala seed and the BOLD response of the pgPFC cluster. Amygdala reactivity and recovery data are calculated as percent signal BOLD change from baseline in each epoch. Likability of faces data were self-reported by participants. ^IAPS^ Denotes the values were taken from the International Affective Picture set standard ratings. IAPS pictures included: Negative images: 1111, 1220, 1275, 1525, 2053, 2205, 2278, 2490, 2700, 2717, 2750, 2799, 2800, 3216, 3280, 5973, 6200, 6210, 6250, 6311, 6562, 6570, 6838, 6840, 7359, 7361, 9000, 9001, 9007, 9008, 9090, 9101, 9140, 9182, 9265, 9280, 9290, 9300, 9301, 9320, 9331, 9373, 9419, 9425, 9426, 9471, 9520, 9561, 9570, 9571, 9584, 9600, 9622, 9630, 9810, 9830, 9902, 9903, 9911, 9925. Neutral images: 1616, 2038, 2102, 2191, 2200, 2210, 2214, 2215, 2305, 2357, 2385, 2393, 2396, 2397, 2441, 2445, 2480, 2487, 2493, 2499, 2512, 2516, 2579, 2595, 2749, 2840, 2850, 5471, 5520, 5535, 6150, 7006, 7009, 7030, 7036, 7037, 7038, 7041, 7044, 7050, 7100, 7130, 7160, 7161, 7170, 7179, 7180, 7184, 7207, 7235, 7242, 7247, 7249, 7484, 7493, 7500, 7547, 7550, 9070, 9700.

## References

[b1] McEwen B. S. . Mechanisms of stress in the brain. Nat. Neurosci. 18(10), 1353–1363 (2015). 26404710 10.1038/nn.4086 PMC4933289

[b2] Burghy C. A. . Developmental pathways to amygdala-prefrontal function and internalizing symptoms in adolescence. Nat. Neurosci. 15(12), 1736–1741 (2012). 23143517 10.1038/nn.3257 PMC3509229

[b3] Schreiber J. E. . Environmental influences on family similarity in afternoon cortisol levels: twin and parent-offspring designs. Psychoneuroendocrino. 31(9), 1131–1137 (2006). 10.1016/j.psyneuen.2006.07.005 PMC2754130 16997489

[b4] Gunnar M. R. & Hostinar C. E. The social buffering of the hypothalamic-pituitary-adrenocortical axis in humans: Developmental and experiential determinants. Soc Neurosci. 10(5), 479–488 (2015). 26230646 10.1080/17470919.2015.1070747 PMC4618716

[b5] Gunnar M. R. & Quevedo K. The neurobiology of stress and development. Annu Rev. Psychol. 58, 145–173 (2007). 16903808 10.1146/annurev.psych.58.110405.085605

[b6] Van Hulle C. A., Shirtcliff E. A., Lemery-Chalfant K. & Goldsmith H. H. Genetic and environmental influences on individual differences in cortisol level and circadian rhythm in middle childhood. Horm Behav. 62(1), 36–42 (2012). 22583671 10.1016/j.yhbeh.2012.04.014 PMC3377812

[b7] Essex M. J., Klein M. H., Cho E. & Kalin N. H. Maternal stress beginning in infancy may sensitize children to later stress exposure: effects on cortisol and behavior. Biol Psychiat. 52, 776–784 (2002). 12372649 10.1016/s0006-3223(02)01553-6

[b8] Urry H. L. . Amygdala and ventromedial prefrontal cortex are inversely coupled during regulation of negative affect and predict the diurnal pattern of cortisol secretion among older adults. J. Neurosci. 26(16), 4415–4425 (2006). 16624961 10.1523/JNEUROSCI.3215-05.2006 PMC6673990

[b9] Veer I. M. . Endogenous cortisol is associated with functional connectivity between the amygdala and medial prefrontal cortex. Psychoneuroendocrino. 37(7), 1039–1047 (2012). 10.1016/j.psyneuen.2011.12.001 22204928

[b10] Gee D. G. . Early developmental emergence of human amygdala-prefrontal connectivity after maternal deprivation. Proc. Nat Acad Sci. 110(39), 15638–15643 (2013). 24019460 10.1073/pnas.1307893110 PMC3785723

[b11] Sanchez M. D., Milanés M. V., Pazos A., Diaz A. & Laorden M. L. Autoradiographic evidence of delta-opioid receptor downregulation after prenatal stress in offspring rat brain. Pharmacology 60(1), 13–18 (2000). 10629438 10.1159/000028341

[b12] Anisman H., Zaharia M. D., Meaney M. J. & Merali Z. Do early-life events permanently alter behavioral and hormonal responses to stressors? Int J. Dev Neurosci. 16 **(3–4)**, 149–164 (1998). 9785112 10.1016/s0736-5748(98)00025-2

[b13] Myers-Schulz B. & Koenigs M. Functional anatomy of ventromedial prefrontal cortex: implications for mood and anxiety disorders. Mol Psychiatr. 17(2), 132–141 (2012). 10.1038/mp.2011.88 PMC3937071 21788943

[b14] Kim M. J., Gee D. G., Loucks R. A., Davis F. C. & Whalen P. J. Anxiety dissociates dorsal and ventral medial prefrontal cortex functional connectivity with the amygdala at rest. Cereb Cortex. 21, 1667–1673 (2011). 21127016 10.1093/cercor/bhq237 PMC3116741

[b15] Kim M. J. . The structural and functional connectivity of the amygdala; from normal emotion to pathological anxiety. Behav Brain Res. 223, 403–410 (2011). 21536077 10.1016/j.bbr.2011.04.025 PMC3119771

[b16] Salomons T. V., Johnstone T., Backonja M. M., Shackman A. J. & Davidson R. J. Individual differences in the effects of perceived controllability on pain perception: critical role of the prefrontal cortex. J. Cogn Neurosci. 19(6), 993–1003 (2007). 17536969 10.1162/jocn.2007.19.6.993

[b17] Compas B. E., Connor-Smith J. K., Saltzman H., Thomsen A. H. & Wadsworth M. E. Coping with stress during childhood and adolescence: problems, progress, and potential in theory and research. Psychol Bul. 127(1), 87–127 (2001). 11271757

[b18] Meyer S., Raikes H. A., Virmani E. A., Waters S. & Thompson R. A. Parent emotion representations and the socialization of emotion regulation in the family. Int J. Behav Dev. 38(2), 164–173 (2014).

[b19] Fraga M. F. . Epigenetic differences arise during the lifetime of monozygotic twins. Proc. Nat Acad Sci. 102(30), 10604–10609 (2005). 16009939 10.1073/pnas.0500398102 PMC1174919

[b20] Lahey B. B. & D’Onofrio B. M. All in the family: comparing siblings to test causal hypotheses regarding environmental influences on behavior. Curr Dir. Psychol Sci. 19(5), 319–323 (2010). 23645975 10.1177/0963721410383977 PMC3643791

[b21] Castellanos F. X. . Anatomic brain abnormalities in monozygotic twins discordant for attention deficit hyperactivity disorder. Am J. Psychiat. 160(9), 1693–1696 (2003). 12944348 10.1176/appi.ajp.160.9.1693

[b22] Van’t Ent D. . A structural MRI study in monozygotic twins concordant or discordant for attention/hyperactivity problems: evidence for genetic and environmental heterogeneity in the developing brain. Neuroimage. 35(3), 1004–1020 (2007). 17346990 10.1016/j.neuroimage.2007.01.037 PMC1939804

[b23] de Geus E. J. . Intrapair differences in hippocampal volume in monozygotic twins discordant for the risk for anxiety and depression. Biol Psychiat. 61(9), 1062–1071 (2007). 17137562 10.1016/j.biopsych.2006.07.026

[b24] Pell G. S. . Reduced variance in monozygous twins for multiple MR parameters: implications for disease studies and the genetic basis of brain structure. Neuroimage. 49(2), 1536–1544 (2010). 19747554 10.1016/j.neuroimage.2009.09.003

[b25] Debiec J. & Sullivan R. M. Intergenerational transmission of emotional trauma through amygdala-dependent mother-to-infant transfer of specific fear. Proc. Nat Acad Sci. 111(33), 12222–12227 (2014). 25071168 10.1073/pnas.1316740111 PMC4142995

[b26] Waszczuk M. A., Zavos H. M. S., Gregory A. M. & Eley T. C. The phenotypic and genetic structure of depression and anxiety disorder symptoms in childhood, adolescence and young adulthood. J. Am Acad. Psychiat. 71(8), 905–916 (2014). 10.1001/jamapsychiatry.2014.655 24920372

[b27] Lang P. J., Bradley M. M. & Cuthbert B. N. International affective picture system (IAPS): Affective ratings of pictures and instruction manual. Technical Report A-8 (University of Florida, Gainesville, FL) (2008).

[b28] Schuyler B. S. . Temporal dynamics of emotional responding: amygdala recovery predicts emotional traits. Soc Cogn Affect Neurosci. 9(2), 176–181 (2014). 23160815 10.1093/scan/nss131 PMC3907933

[b29] Carver C. S., Scheier M. F. & Weintraub J. K. Assessing coping strategies: a theoretically based approach. J. Pers. Soc Psychol. 56(2), 267–283 (1989). 2926629 10.1037//0022-3514.56.2.267

[b30] Miskovic V. & Schmidt L. A. Social fearfulness in the human brain. Neurosci Biobehav. Rev 36(1), 459–478 (2012). 21855571 10.1016/j.neubiorev.2011.08.002

[b31] Freund J. . Emergence of individuality in genetically identical mice. Science. 340(6133), 756–759 (2013). 23661762 10.1126/science.1235294

[b32] Goldsmith H. H., Lemery-Chalfant K., Schmidt N. L., Arneson C. L. & Schmidt C. K. Longitudinal analyses of affect, temperament, and childhood psychopathology. Twin Res Hum Genet. 10, 118–126 (2007). 17539371 10.1375/twin.10.1.118

[b33] Cox R. W. AFNI: software for analysis and visualization of functional magnetic resonance images. Comput Biomed Res. 29, 162–173 (1996). 8812068 10.1006/cbmr.1996.0014

[b34] Saad Z. S. . Trouble at rest: how correlation patterns and group differences become distorted after global signal regression. Brain Connect. 2(1), 25–32 (2009). 10.1089/brain.2012.0080 PMC3484684 22432927

[b35] Ashburner J. A fast diffeomorphic image registration algorithm. Neuroimage. 38(1), 95–113 (2007). 17761438 10.1016/j.neuroimage.2007.07.007

[b36] Smith S. M. . Advances in functional and structural MR image analysis and implementation as FSL. Neuroimage. 23, S208–S219 (2004). 15501092 10.1016/j.neuroimage.2004.07.051

[b37] Woolrich M. W. . Bayesian analysis of neuroimaging data in FSL. Neuroimage. 45, S173–S186 (2009). 19059349 10.1016/j.neuroimage.2008.10.055

[b38] Weissenbacher A. . Correlations and anticorrelations in resting-state functional connectivity MRI: a quantitative comparison of preprocessing strategies. Neuroimage. 47, 1408–1416 (2009). 19442749 10.1016/j.neuroimage.2009.05.005

[b39] Cordes D. . Frequencies contributing to functional connectivity in the cerebral cortex in “resting-state” data. AJNR Am J. Neuroradiol. 22, 1326–1333 (2001). 11498421 PMC7975218

[b40] Biswal B., Zerrin Yetkin F., Haughton V. M. & Hyde J. S. Functional connectivity in the motor cortex of resting human brain using echo-planar MRI. Magn Reson Med. 34, 537–541 (1995). 8524021 10.1002/mrm.1910340409

[b41] Lancaster J. L. . Automated Talairach Atlas labels for functional brain mapping. Hum Brain Mapp. 10, 120–131 (2000). 10912591 10.1002/1097-0193(200007)10:3<120::AID-HBM30>3.0.CO;2-8 PMC6871915

[b42] Tottenham N. . The NIMSTIM set of facial expressions: judgments from untrained research participants. Psychiatry Res. 168(3), 242–249 (2009). 19564050 10.1016/j.psychres.2008.05.006 PMC3474329

[b43] Messer K., Matas J., Kittler J., Luettin J. & Maitre G. “XM2VTSdb: The Extended M2VTS Database, Proceedings 2^nd^ Conference on Audio and Video-base Biometric Personal Verification (AVBPA99)” (Springer Verlag, New York), http://www.ee.surrey.ac.uk/Research/VSSP/sm2vtsdb (1999).

[b44] Lundqvist D., Flykt A. & Öhman A. The Karolinska Directed Emotional Faces–KDEF, CD ROM from Department of Clinical Neuroscience, Psychology section, Karolinska Instituet: ISBN 91-630-7164-9 (1998).

